# Configuration-Selective Photocurrent Enhancement Induced by Static Domain Walls in Two-Dimensional Ferroelectric In_2_Se_3_

**DOI:** 10.3390/nano16110682

**Published:** 2026-06-01

**Authors:** Ning Xu, Yuehua Xu

**Affiliations:** School of Wang Zheng Microelectronics, Changzhou University, Changzhou 213164, China; s23060809028@smail.cczu.edu.cn

**Keywords:** two-dimensional ferroelectric materials, static domain walls, configuration-selective, In_2_Se_3_, first-principles calculations, photocurrent enhancement

## Abstract

Domain walls (DWs) are ubiquitous topological defects in two-dimensional (2D) ferroelectric materials, yet their static role in optoelectronic transport remains unclear. Here, we address this issue using first-principles quantum-transport calculations on monolayer ferroelectric In_2_Se_3_ p–i–n junctions. Contrary to the conventional view that defects degrade device performance, only specific static DW configurations—not all—can significantly enhance photocurrent. We examine two thermodynamically stable configurations (the Initial and Final states) and one saddle-point configuration (the Transition state) along the polarization-switching pathway. The Initial state yields a photocurrent density of 10.91 μA·mm^−2^, about 1.80 times that of the single-domain device, while the Final state reaches 8.39 μA·mm^−2^, corresponding to an increase of ~37%. By comparison, the thermodynamically unstable Transition state gives a lower value of 5.92 μA·mm^−2^, indicating strong configuration selectivity. Analysis shows that the observed behavior can be qualitatively rationalized by the combined effects of optical absorption, carrier separation induced by DW-driven electrostatic-potential redistribution, and preserved conduction-channel continuity for carrier extraction. These findings provide a microscopic basis for understanding configuration-selective photocurrent enhancement by static domain walls in short-channel 2D ferroelectric devices.

## 1. Introduction

Polar materials have emerged as transformative components in advanced semiconductor devices. Within this broad family, while antiferroelectric materials are currently driving innovations in reconfigurable neuromorphic hardware leveraging their volatile switching dynamics [1], ferroelectric materials—distinguished by stable spontaneous polarization and non-volatile domains—offer distinct advantages for modulating optoelectronic transport. However, while the dynamic switching kinetics of two-dimensional (2D) ferroelectrics have been extensively studied, the static role of stable domain walls in optoelectronic transport remains poorly understood. Ferroelectric materials, characterized by spontaneous polarization that can be reversed under an external electric field [2,3,4,5], play a central role in both fundamental condensed-matter physics and device applications. The coupling between ferroelectric polarization and electronic transport has enabled the development of non-volatile memory devices [6,7,8], neuromorphic computing systems [9,10], and tunable optoelectronic sensors [11]. As device dimensions approach the nanoscale, two-dimensional (2D) van der Waals (vdW) [12,13] ferroelectrics have emerged as a promising platform for exploring low-dimensional polarization physics. Among them, indium selenide (In_2_Se_3_) [14,15,16,17,18,19,20], particularly in its zincblende-derived phase (ZB′), is a prototypical ferroelectric semiconductor. It exhibits robust room-temperature ferroelectricity down to the monolayer limit and strong light–matter interaction, making it highly suitable for self-powered photodetectors.

The macroscopic performance of ferroelectric devices—from hysteresis behavior to photovoltaic conversion efficiency [21,22]—is governed by the spatial distribution of polar domains and their boundaries, namely domain walls (DWs) [23,24,25,26,27]. Traditionally, DWs have been regarded as structural defects or scattering centers. However, recent studies have highlighted their dynamic behavior, showing that DW-mediated switching pathways exhibit significantly lower activation energy barriers (0.28–0.40 eV per formula unit) than the bulk lattice rotation (0.85 eV per formula unit), thereby dominating polarization switching kinetics [28,29,30].

Most existing studies focus on the dynamic behavior of DWs, including their migration and switching mechanisms under external fields. In contrast, the static role of DWs—once stabilized within the device channel—remains poorly understood. When a DW is immobilized in the active region, it introduces a complex interplay between the local electrostatic potential, which drives carrier separation, and the quantum connectivity of electronic states, which governs carrier transport. It therefore remains unclear whether a static DW acts as a recombination center that degrades performance or as a functional interface that enhances carrier separation and collection. Furthermore, symmetry breaking and charge redistribution at DWs can give rise to phenomena absent in the bulk, such as enhanced local conductivity [31,32] and anomalous photovoltaic effects [33,34,35,36]. These findings suggest a paradigm shift from viewing DWs as defects to considering them as functional units for device optimization [37]. Nevertheless, a microscopic and quantitative understanding of the static effects of thermodynamically stable DW configurations on optoelectronic transport is still lacking. In particular, most theoretical studies rely on ideal single-domain models, neglecting the inevitable presence of DWs in realistic devices. This limitation hinders the development of domain-wall engineering strategies in optoelectronics.

In this work, we perform first-principles quantum-transport calculations on monolayer ZB′-In_2_Se_3_ p–i–n junctions. Two thermodynamically stable DW configurations (Initial and Final states) along the polarization-switching pathway are investigated, with the saddle-point configuration (Transition state) used as a reference. We find that both stable DW configurations significantly enhance the photocurrent, whereas the Transition state exhibits the weakest response, indicating strong configuration selectivity. The enhancement in the Initial state reaches ~78%. We show that the enhancement can be qualitatively rationalized in terms of a balanced interplay among optical absorption (effective density of transition-relevant states), carrier separation (spatial distribution of the built-in potential), and carrier extraction (quantum connectivity of conduction-band states). For a 50 Å short-channel p–i–n junction, the device containing the Initial-state DW achieves a photocurrent density of 10.91 μA·mm^−2^, approximately 1.80 times that of the single-domain device (6.13 μA·mm^−2^). This improvement is attributed to DW-induced modulation of the local potential, which enhances carrier separation while preserving the continuity of conduction pathways required for efficient transport. This short-channel model is used here as a representative platform for identifying configuration-selective static DW effects, rather than for establishing a universal channel-length scaling law or quantitative independence from the chosen doping profile. These results provide a microscopic understanding of how static DW configurations influence photocurrent and highlight their potential as functional building blocks for short-channel 2D optoelectronic devices.

## 2. Materials and Methods

### 2.1. Calculation Methods for Geometry and Electronic Properties of Monolayer ZB′-In_2_Se_3_

Geometry optimizations and electronic structure calculations of monolayer ferroelectric ZB′-In_2_Se_3_ were performed using the QuantumATK (version S-2022.12, Synopsys, Mountain View, CA, USA) package based on DFT [38,39,40,41,42,43,44,45,46]. The calculations employed the Perdew–Burke–Ernzerhof (PBE) method within the generalized gradient approximation (GGA) [47]. The linear combination of atomic orbitals (LCAO) method [48], and electron-ion interactions were treated with the PseudoDojo pseudopotential [49]. To ensure numerical convergence, the density-mesh cutoff energy was set to 105 Hartree, and the first Brillouin zone [50] was sampled using a Γ-centered 8 × 8 × 1 Monkhorst-Pack k-point grid. Structural relaxation was performed until the residual forces on all atoms were below 0.01 eV·Å^−1^. A vacuum region of ~30 Å was introduced along the surface-normal direction to suppress artificial periodic-image electrostatic interactions, consistent with previous first-principles studies on bi-NiI_2_/In_2_Se_3_ van der Waals heterostructures [51]. Although PBE tends to underestimate the band gap, it reliably describes qualitative trends in electron transport. To validate the electronic-structure description against available experimental data, we further calculated the band structure and band gap using the Heyd–Scuseria–Ernzerhof (HSE06) hybrid functional [52,53]. The HSE06 result was used as a benchmark for the intrinsic electronic band gap, whereas the production NEGF photocurrent calculations were performed at the PBE level to maintain computational consistency among all device configurations.

To evaluate the possible influence of spin–orbit coupling (SOC), additional PBE + SOC benchmark calculations were performed for the representative ABBCA single-domain configuration, because In and Se are relatively heavy elements and SOC may affect band-edge splitting and optical transitions. The optical absorption spectra calculated with and without SOC were compared to assess whether SOC modifies the dominant optical-transition features relevant to the photocurrent response. In the benchmark comparison, the two spectra are nearly superposed in the relevant photon-energy range. The absorption onset, main peak positions, and overall spectral profile remain essentially unchanged after SOC is included, and no additional SOC-induced absorption feature is observed. These results indicate that SOC may only slightly renormalize the absolute electronic energies, but does not qualitatively modify the dominant optical-transition characteristics of monolayer ZB′-In_2_Se_3_. This conclusion is consistent with previous first-principles studies on α-In_2_Se_3_-based ferroelectric devices, where the band structure of monolayer α-In_2_Se_3_ was reported to remain almost unchanged after SOC was included [54]. Therefore, the production NEGF photocurrent calculations were performed without explicit SOC for computational consistency among all domain-wall configurations, while the SOC benchmark confirms the robustness of the electronic and optical trends. Since all compared devices have the same chemical composition and differ mainly in static domain-wall geometry, the configuration-dependent photocurrent trends discussed below are governed primarily by domain-wall-induced electrostatic-potential redistribution and conduction-channel connectivity rather than by SOC-driven band splitting. Additionally, the minimum energy path and activation barrier for domain wall (DW) migration were determined using the climbing image nudged elastic band (CI-NEB) method [55].

### 2.2. Method for Photocurrent Calculation

The photocurrent density of the device was simulated using the non-equilibrium Green’s function (NEGF) method combined with first-order perturbation theory, employing the first Born approximation [56,57]. The interaction between electrons and the light field, treated as a perturbation, is described by the following Hamiltonian:

(1) $$\hat{H}={\hat{H}}_{0}+\frac{e}{m_{0}}A\cdot \hat{p}$$

where ${\hat{H}}_{0}$ is the Hamiltonian of the two-probe system, *e* is the elementary charge, $m_{0}$ is the free-electron mass, $\hat{p}$ is the momentum operator of the electron, and A is the vector potential of the electromagnetic field. Based on this perturbation, the light-induced transmission coefficient $T_{\alpha }(E)$ into electrode α (left *L* or right *R*) is given by [58]:

(2) $$T_{\alpha }\left(E\right)=Tr\left\{i\Gamma_{\alpha }\left[\left(1-f_{\alpha }\right)G_{ph}^{<}+f_{\alpha }G_{ph}^{>}\right]\right\}$$

where $\Gamma_{\alpha }(E)$ is the broadening matrix describing the coupling between the central scattering region and electrode α (left *L* or right *R*); $f_{\alpha }(E)$ represents the Fermi–Dirac distribution function of the electrode at the chemical potential $\mu_{\alpha }$; $G_{ph}^{<}$ and $G_{ph}^{>}$ denote the first-order corrections to the lesser and greater Green’s functions, respectively, arising from photon interactions in the Keldysh formalism. Finally, the photocurrent density $J_{\mathrm{ph}}$ is calculated by integrating the transmission coefficient over the energy spectrum [59]:

(3) $$J_{ph}=\frac{e}{S\hbar }\int \frac{dE}{2\pi }\sum_{\alpha }T_{\alpha }\left(E\right)$$

where e is the electron charge, S is the lateral cross-sectional area of the device, and ℏ is the reduced Planck’s constant. The integral term represents the summation over the energy E, accounting for the contribution of electron transmission at different energy states.

The real-space local density of states (LDOS) was obtained from the self-consistent two-probe NEGF calculation, rather than from an isolated periodic monolayer calculation. In the NEGF formalism, the retarded Green’s function of the open device is expressed as:

(4) $$G\left(\varepsilon \right)={\left[\left(\varepsilon +i\delta_{+}\right)S-H-\sum^{L}\left(\varepsilon \right)-\sum^{R}\left(\varepsilon \right)\right]}^{-1}$$

where H is the Hamiltonian of the central scattering region, S is the overlap matrix in the LCAO basis, and $\sum^{L/R}\left(\varepsilon \right)$ are the retarded self-energies of the left and right electrodes [60]. The spectral density matrix was then projected onto the real-space LCAO basis to obtain the LDOS:

(5) $$D\left(E,r\right)=\sum_{ij}\rho_{ij}(E)\varphi_{i}(r)\varphi_{j}\left(r\right)$$

where $\rho_{ij}(E)$ is the spectral density matrix and $\varphi_{i}(r)$ and $\varphi_{j}(r)$ are localized atomic orbitals. Finally, the LDOS map along the transport direction was obtained by cross-sectionally averaging $D\left(E,r\right)$ over the transverse directions, yielding LDOS(*z*, *E*). Therefore, the spatial distribution of electrode-coupled available states in the open p–i–n device was analyzed. Regions with strongly depleted electrode-coupled LDOS suggest possible transport bottlenecks, where carrier propagation and transmission are expected to be suppressed.

It should be emphasized that the photocurrent calculations were performed at the PBE level. Although PBE tends to underestimate the intrinsic band gap, the device in this study operates under high-doping conditions, where the high carrier concentration significantly enhances electrostatic screening and suppresses electron–hole interactions. As a result, many-body effects such as GW quasiparticle corrections and exciton binding are expected to be strongly reduced. Previous studies of similar two-dimensional (2D) optoelectronic devices have shown that photocurrent spectra computed at the PBE level are qualitatively consistent with experimental results [61,62]. More importantly, since the present work focuses on configuration-dependent trends rather than absolute values, the relative comparison among different domain-wall configurations is expected to be robust with respect to the choice of exchange–correlation functional. We further benchmark the band gap using the HSE06 functional to provide a more accurate reference for the electronic structure. Overall, PBE offers a computationally tractable and physically reasonable approach for capturing configuration-dependent photocurrent trends in this study.

## 3. Results and Discussion

### 3.1. Geometric Structure and Electronic Properties of Monolayer ZB′-In_2_Se_3_

**Figure 1:**
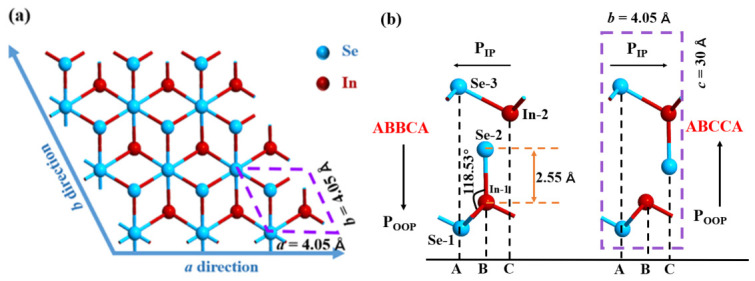
Ferroelectric ZB′-In_2_Se_3_: (**a**) top view and (**b**) side views of the two oppositely polarized stacking structures. Here, *a* and *b* are the in-plane lattice constants, *c* is the lattice constant along the vacuum direction, and the primitive cell is outlined in purple. The black arrows in (**b**) indicate the direction of spontaneous polarization (*P*), and the yellow dashed line marks the bond length between Se-2 and In-1.

**Figure 2:**
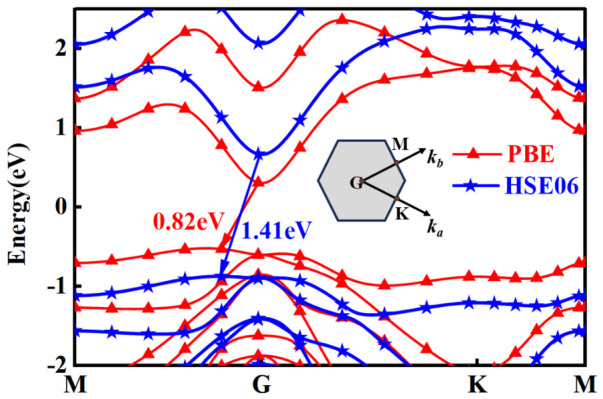
Band structures of monolayer ZB′-In_2_Se_3_ calculated using the PBE and HSE06 functionals. The inset shows the first Brillouin zone. Triangular curves represent PBE results, while pentagonal curves represent HSE06 results.

Monolayer ferroelectric ZB′-In_2_Se_3_ adopts a vertically stacked five-atomic-layer sequence: Se-In-Se-In-Se (Figure 1a) [63]. After relaxation, the in-plane lattice constant is 4.05 Å, in excellent agreement with the experimental value of 4.00 Å [64,65]. The bond length between Se-2 and In-1 is 2.55 Å, and the bond angle between In-1 and Se-1(2) is 118.53°, both consistent with previous theoretical results (Figure 1b). The ferroelectric polarization arises from asymmetric interlayer coordination. The vertical displacement of the middle Se layer relative to the upper and lower layers breaks the out-of-plane mirror symmetry. When the central Se atom occupies the B site (ABBCA stacking), the out-of-plane polarization points downward; when it moves to the C site (ABCCA stacking), the polarization reverses upward (Figure 1b). The PBE functional yields a band gap of 0.82 eV (Figure 2), which increases to 1.41 eV when calculated with the HSE06 functional, in good agreement with the experimentally reported value of 1.46 eV [63].

Since polarization reversal is driven by a sub-angstrom displacement of a single atomic layer, the domain wall—representing the spatial transition between the two stacking sequences—only involves highly localized atomic rearrangements. Consequently, variations in the local coordination environment directly influence the electronic structure and transport properties in the domain wall region.

**Figure 3:**
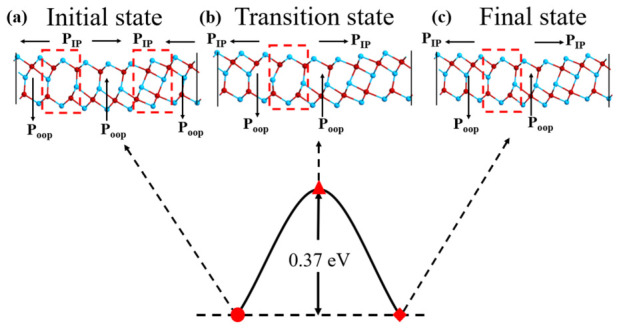
Domain-wall configurations along the polarization-switching pathway in monolayer In_2_Se_3_: (**a**) Initial state with two domain walls (DWs), (**b**) Transition state with one DW, and (**c**) Final state with one DW located between two ferroelectric domains of opposite polarization. The corresponding energy profile along the switching pathway is shown below. Red boxes indicate the positions of the DWs.

Polarization reversal occurs via domain wall (DW)-assisted migration. The climbing-image nudged elastic band (CI-NEB) calculation reveals the potential energy landscape along the switching path (Figure 3). The Initial state (Figure 3a) and Final state (Figure 3c) correspond to two local minima, representing thermodynamically stable DW configurations that can persist in the device channel at room temperature. Therefore, in real optoelectronic devices operating under steady-state conditions where active polarization switching is absent, these DWs are not highly dynamic but are inevitably immobilized within the active channel. Their static presence directly relates to real device performance by acting as functional interfaces that redistribute the built-in potential and modulate steady-state carrier separation. In contrast, the Transition state (Figure 3b) lies at the saddle point between them and corresponds to a transient configuration. These three states provide a natural basis for a configuration-dependent comparison in this study. By comparing the optoelectronic performance of the two thermodynamically stable configurations with that of the saddle-point configuration, we systematically evaluate how sensitive the device response is to the atomic structure of the DW. The CI-NEB calculation identifies two switching paths with slightly different barriers, but both exhibit the same qualitative trend in the modulation of photocurrent by DW configuration. In this work, we focus on the representative path with a barrier of 0.37 eV.

### 3.2. Photocurrent Characteristics and Physical Origin in Single-Domain In_2_Se_3_ Devices

**Figure 4:**
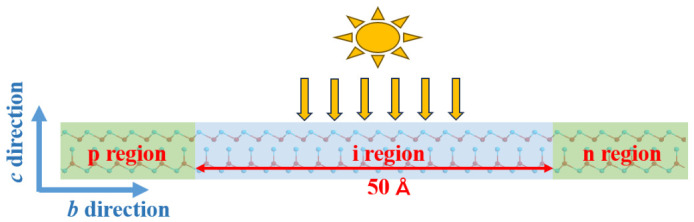
Schematic illustration of the p–i–n junction based on monolayer ZB′-In_2_Se_3_ with the ABBCA stacking configuration. The length of the central intrinsic (i) region is 50 Å.

We constructed a two-probe model based on a photovoltaic p–i–n junction [66], using the ABBCA configuration as an example, as shown in Figure 4. The structure consists of a p-doped region, an intrinsic (i) region, and an n-doped region. The length of the i region is 50 Å. The specific doping concentrations (10^21^ cm^−3^ for p-type, 10^19^ cm^−3^ for n-type) are not arbitrary [67,68,69]. They are determined by the requirement that, in the p-type electrode, the valence band maximum (VBM) lies 0.15 eV above the Fermi level, and in the n-type electrode, the conduction band minimum (CBM) lies 0.15 eV below the Fermi level [70], combined with the different effective densities of states of the valence and conduction bands in monolayer ZB′-In_2_Se_3_. And the doping concentrations may be achieved in experiments. Although using the same doping profile for all devices ensures a controlled comparison in which the DW configuration is the only explicit structural variable, it does not by itself exclude the possibility that the chosen asymmetric doping condition may amplify or suppress the quantitative magnitude of the observed DW-induced enhancement. Therefore, the present trends should be understood as configuration-dependent results established within a fixed doping scheme, rather than as proof that the observed enhancement is fully independent of doping asymmetry. Accordingly, the present work establishes configuration-selective DW effects within a fixed asymmetric p–i–n doping scheme, rather than demonstrating that the quantitative magnitude of the enhancement is universal with respect to arbitrary doping profiles. Since polarized light is incident vertically on the scattering region rather than on the electrodes, the i region—the core of the device—serves as the primary photon-absorption region and efficiently captures incident photons. This p–i–n architecture provides an ideal computational platform for studying the effects of DWs on optoelectronic transport: the intrinsic region hosts both photon absorption and carrier transport. When a DW is embedded in the i region, its modulation of the local electronic structure, electrostatic potential distribution, and transport connectivity is directly reflected in the measurable photocurrent. Meanwhile, the built-in electric field generated by the highly doped p/n regions extends across the i region and drives the separation and directional transport of photogenerated carriers. This provides a useful framework for qualitatively analyzing the respective roles of the DW in absorption, carrier separation, and carrier extraction within a unified self-consistent picture.

#### 3.2.1. Intrinsic Anisotropy and Transport Bottlenecks in Single-Domain Devices

**Figure 5:**
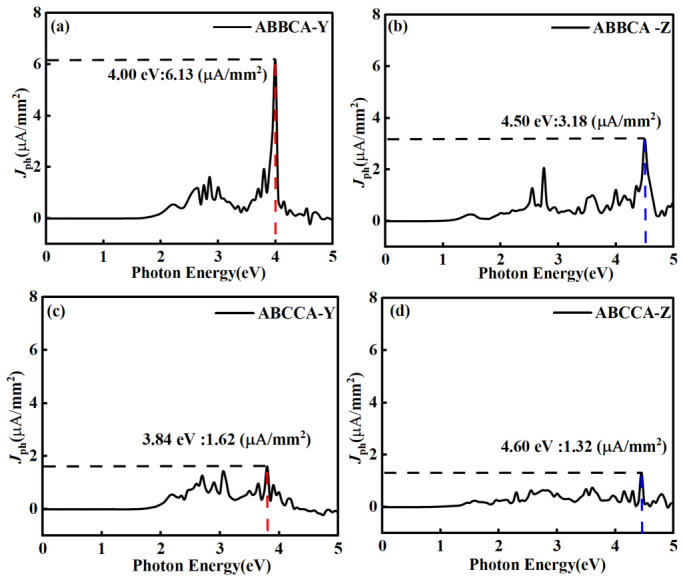
Photocurrent-density spectra *J*_ph_ of single-domain stackings: (**a**,**b**) ABBCA and (**c**,**d**) ABCCA under *y*-polarized (out-of-plane) and *z*-polarized (in-plane transport direction) light.

To evaluate the modulation of optoelectronic transport by domain walls, we first constructed spatially uniform single-domain p–i–n junctions as benchmark systems. We examined the intrinsic photocurrent of the ABBCA and ABCCA stackings. These single-domain benchmarks serve two purposes: they provide a quantitative reference for subsequent changes induced by DWs and reveal the intrinsic transport limitations of the single-domain systems, offering a physical starting point for understanding how DW configurations modify these limitations. As shown in Figure 5, the photocurrent response is strongly dependent on the polarization state. Under *y*-polarized light (out-of-plane), the ABBCA stacking exhibits a peak photocurrent density of 6.13 μA·mm^−2^ at a photon energy of 4.14 eV. In contrast, the peak photocurrent of the ABCCA stacking is only 1.62 μA·mm^−2^, nearly three-quarters lower than that of ABBCA. This pronounced polarization dependence cannot be explained solely by optical selection rules, suggesting that polarization reversal significantly alters the spatial distribution of electronic states in the channel—a conclusion further supported by the local density of states analysis.

**Figure 6:**
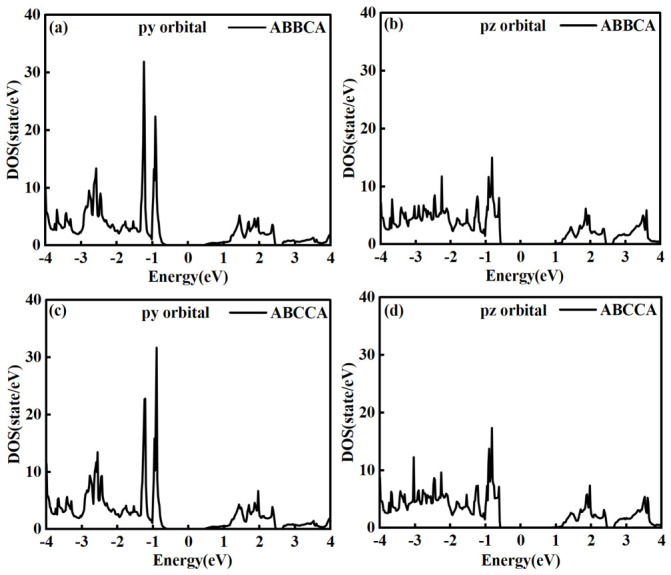
Orbital-resolved projected density of states (PDOS) for the single-domain stackings: (**a**,**b**) ABBCA and (**c**,**d**) ABCCA.

The ABBCA stacking exhibits strong optical anisotropy [71] (*J*_ph,y_ >> *J*_ph,z_), which originates from the dominance of *p*_y_ orbitals at the band edges, as shown in Figure 6. This leads to much stronger coupling to out-of-plane polarized light than to in-plane polarized light. In contrast, in the ABCCA stacking, this intrinsic anisotropy nearly disappears, replaced by a weak and nearly isotropic photocurrent. This anomalous behavior suggests that ABCCA is limited by a more fundamental mechanism than optical selection rules: once the transport channel is severely blocked, the absorption anisotropy is overwhelmed by the transport bottleneck during the cascade from excitation to collection, preventing it from influencing the macroscopic photocurrent.

**Figure 7:**
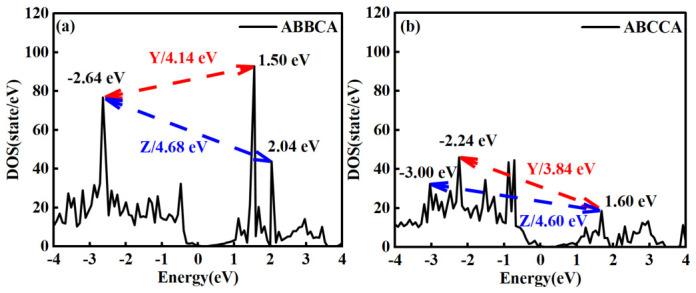
Total density of states (DOS) in the device channel for the single-domain stackings: (**a**) ABBCA and (**b**) ABCCA, calculated using the PBE functional.

This suppression does not arise solely from an insufficient density of available states. As shown by the density-of-states (DOS) analysis (Figure 7), the DOS of the ABCCA stacking is indeed lower, thereby weakening its intrinsic optical absorption to some extent. However, the photocurrent magnitude is not determined solely by optical excitation; it results from the cumulative effects of optical absorption, photocarrier separation, and microscopic carrier extraction (quantum connectivity). This motivates us to analyze the downstream processes.

**Figure 8:**
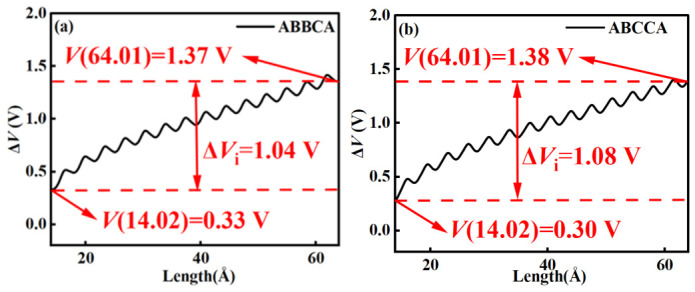
Electrostatic potential profiles along the transport direction in the intrinsic region of the single-domain devices: (**a**) ABBCA stacking and (**b**) ABCCA stacking. Red dashed lines indicate the potential values at the p- and n-electrode boundaries.

To examine carrier separation, we calculated the macroscopic electrostatic potential profile in the intrinsic region of the single-domain devices. As shown in Figure 8, although this crystal phase intrinsically hosts interlocked in-plane (IP) and out-of-plane (OOP) spontaneous polarizations, the two single-domain configurations, ABBCA and ABCCA, exhibit nearly identical total potential drops across the channel (1.04 V and 1.08 V, respectively), with a uniform linear decay throughout the intrinsic region. This macroscopic similarity directly rules out differences in the built-in field as the cause of photocurrent suppression. More importantly, it reveals a key screening mechanism in monolayer ZB′-In_2_Se_3_ transport: in the highly doped p–i–n geometry considered here, the total potential drop is dominated by the doping gradient at the two ends. This finding is consistent with the results of Fang et al. [68] for analogous α-In_2_Se_3_ p–i–n devices, where the two single-domain configurations provide nearly identical macroscopic driving fields for carrier separation. In the next section, we examine whether abrupt local atomic reconstruction at a DW modifies the spatial potential distribution and thereby gives rise to configuration-dependent optoelectronic transport in DW-containing devices.

**Figure 9:**
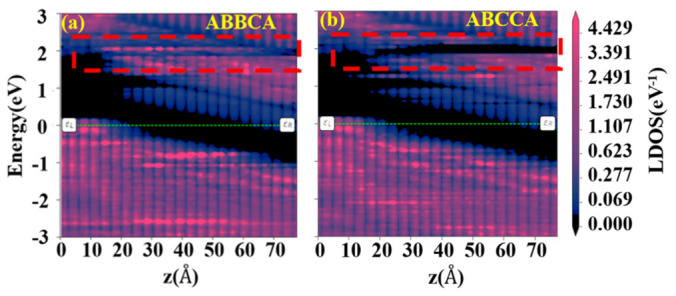
LDOS(*z*, *E*) for the two-probe configurations of the (**a**) ABBCA and (**b**) ABCCA devices along the transport direction *z*. The LDOS was obtained from the retarded Green’s function of the self-consistent NEGF calculation by projecting the spectral density onto the real-space grid and integrating/averaging over the transverse directions. The energy is referenced to the Fermi level. Bright regions indicate spatially continuous electrode-coupled states, whereas dark regions indicate depleted LDOS and possible transport bottlenecks.

We now turn to the microscopic process of carrier extraction. The LDOS resolved along the transport direction reveals a key difference (Figure 9): in the ABBCA stacking, conduction-band states remain spatially continuous throughout the intrinsic region (Figure 9a), allowing carriers to travel along the conduction channel from the absorption region to the n electrode. In contrast, in the ABCCA stacking, the conduction-band states are highly fragmented in real space (Figure 9b) and appear as a series of isolated localized states. Although these localized states contribute to the DOS, they cannot support long-range carrier transport.

Within the NEGF framework, an LDOS approaching zero at a given position means that the real-space propagation of the Green’s function is cut off, and the transmission probability is exponentially suppressed. Regardless of how many photogenerated carriers are created upstream, they cannot traverse these LDOS-depleted regions to reach the electrode. Therefore, the fragmented conduction-band states in the ABCCA stacking fundamentally limit photocurrent collection. In the following analysis of DW-containing devices, we examine how the DW configuration modulates optical absorption, carrier separation, and conduction-band connectivity.

#### 3.2.2. Enhancement of Photocurrent by Static Domain Walls

On this basis, we examine how the optoelectronic performance evolves when a DW is introduced into the device channel. From the perspective of transport physics, atomic rearrangement at a DW introduces additional potential scattering, which would conventionally be expected to degrade carrier transport. Meanwhile, the local bound charge associated with polarization discontinuity redistributes the electrostatic potential, thereby modifying the driving force for carrier separation. Owing to the competition between these effects, the overall impact of a DW on photoelectric performance cannot be predicted a priori.

**Figure 10:**
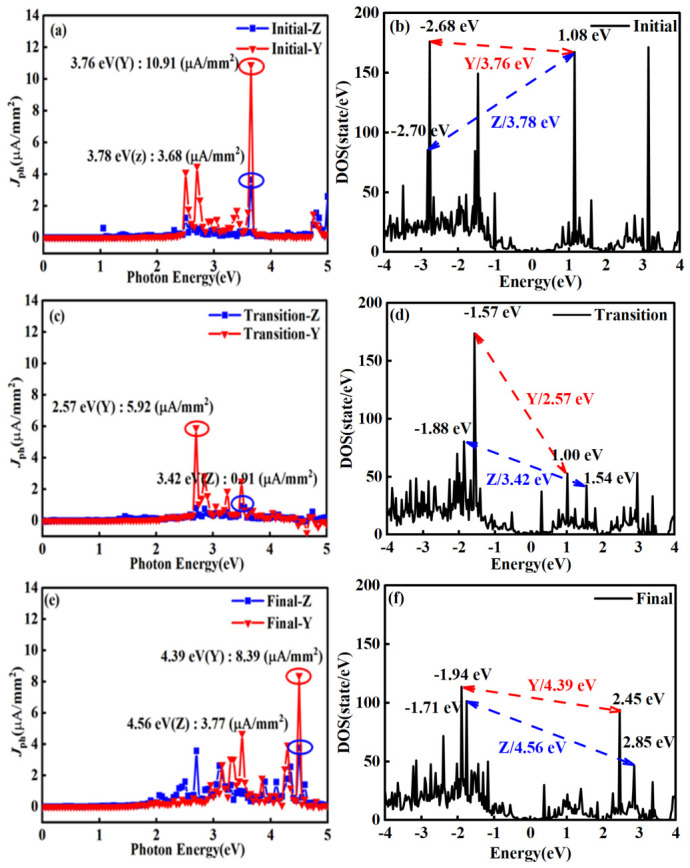
Photocurrent spectra of the device under *z*-polarized (in-plane) and *y*-polarized (out-of-plane) light for the three domain-wall configurations: (**a**,**b**) Initial state, (**c**,**d**) Transition state, and (**e**,**f**) Final state. The corresponding density of states (DOS) in the central channel is shown for each configuration.

To address this issue, we consider two thermodynamically stable configurations on the CI-NEB energy landscape (the Initial and Final states), together with the saddle-point configuration (the Transition state), and embed them in the intrinsic region of the p–i–n junction for photocurrent calculations. The results deviate from the conventional expectation of DWs as scattering centers. Both thermodynamically stable configurations significantly enhance the photocurrent rather than suppressing it. To improve clarity, the photocurrent spectra and the corresponding channel DOS are presented together for each DW configuration, allowing the dominant optical-transition energies to be directly compared with the available valence- and conduction-side states. As shown in Figure 10, all DW-containing devices exhibit much stronger responses under *y*-polarized light than under *z*-polarized light. This behavior is consistent with the single-domain analysis (Section 3.2.1), where the dominant regions of the channel retain a well-defined polarization direction, with the *p*_y_ orbital character at the band edges favoring coupling with out-of-plane polarized light. Although the DW locally breaks symmetry, its spatial extent is insufficient to alter the overall polarization selectivity. Therefore, the following discussion focuses on the *y*-polarized response.

Under *y*-polarized illumination, both stable DW configurations outperform the single-domain benchmark (ABBCA, 6.13 μA·mm^−2^). The Initial state reaches 10.91 μA·mm^−2^ at 3.76 eV, corresponding to an increase of ~78%, while the Final state reaches 8.39 μA·mm^−2^ at 4.39 eV (an increase of ~37%). Notably, the peak of the Final state shifts toward higher photon energy, and the origin of this shift is discussed in Section 3.2.3. In contrast, the saddle-point Transition state (5.92 μA·mm^−2^) shows no appreciable enhancement and remains comparable to the single-domain case. Given that this study focuses on configuration-dependent trends within the same computational setup, the small difference (~3%) does not change the main conclusion regarding configuration selectivity.

These results indicate that photocurrent enhancement is strongly configuration-dependent: not all DW configurations are beneficial. We note that the switching-path configurations compared here differ not only in local atomic arrangement but also in the number of DW segments within the 50 Å channel. Accordingly, the term “configuration selectivity” in the present work refers to the full static DW configuration realized along the switching pathway, rather than to an isolated comparison performed at a strictly fixed DW number. While the unstable transition state yields only marginal performance, specific thermodynamically stable configurations lead to substantial enhancement. The microscopic origin of this configuration selectivity is analyzed below by examining the contributions of optical absorption (effective density of transition-relevant states), carrier separation (spatial distribution of the built-in potential), and carrier extraction (connectivity of conduction-band states).

#### 3.2.3. Microscopic Mechanism of Photocurrent Enhancement: A Three-Factor Analysis of Absorption, Separation, and Connectivity

Before proceeding, we emphasize that the three factors discussed below—optical absorption (DOS), carrier separation (electrostatic potential distribution), and carrier extraction (conduction-band connectivity)—are not independent in the fully self-consistent NEGF framework. The electrostatic potential affects the LDOS, which in turn influences the spatial distribution of charge and the effective absorption region. The following analysis should therefore be understood as a qualitative interpretative framework that decomposes the self-consistently obtained results into three physically intuitive contributions, rather than a rigorous decoupling of independent variables. The quantitative photocurrent values reported in this work are obtained directly from the full NEGF calculations without any decoupling approximation.

With this caveat in mind, we analyze the microscopic origin of the enhanced photocurrent by examining three key stages—optical absorption, carrier separation, and carrier extraction—in terms of the effective density of transition-relevant states, the spatial distribution of the electrostatic potential, and the quantum connectivity of conduction-band states, respectively.

(i) Effective density of transition-relevant states

Efficient photocurrent generation requires a sufficient number of electronic states to support optical transitions. In p–i–n devices, photon absorption mainly occurs in the intrinsic region and is governed by the joint DOS between the valence and conduction bands. By modifying the local electronic structure, different DW configurations directly alter the number of available transition channels.

Figure 10b,d,f show the total DOS in the channel for the three devices containing DWs. For the Initial state, the dominant transition (*ħω* = 3.76 eV) connects two high-DOS peaks located at −2.68 eV (valence band) and 1.08 eV (conduction band), with densities of 176 and 168 states·eV^−1^, respectively. Both values are the highest among the three configurations, favoring efficient optical transitions.

In the Transition state, although the valence-side DOS (174 states·eV^−1^) is comparable to that in the Initial state, the conduction-side DOS is only ~53 states·eV^−1^, less than one-third of the corresponding value in the Initial state. This severe shortage of final states imposes a bottleneck on optical absorption.

For the Final state, the DOS values on the two sides (114 and 94 states·eV^−1^) are intermediate. Its dominant transition occurs at a higher photon energy (4.39 eV), reflecting the relative scarcity of available states near the conduction-band minimum. Consequently, carriers must be excited to higher energies to access a sufficient number of transition channels.

Overall, in terms of effective density of transition-relevant states, the Initial state exhibits the most favorable valence–conduction DOS pairing, the Final state is intermediate, whereas the Transition state is severely limited by the lack of conduction-side final states.

(ii) Carrier-separation efficiency: spatial distribution of the electrostatic potential

In contrast to the nearly uniform potential profile observed in single-domain devices (Section 3.2.1), the bound charge associated with polarization discontinuity at the DW redistributes the built-in potential. This leads to significant variations in the driving force for carrier separation across different regions of the channel.

**Figure 11:**
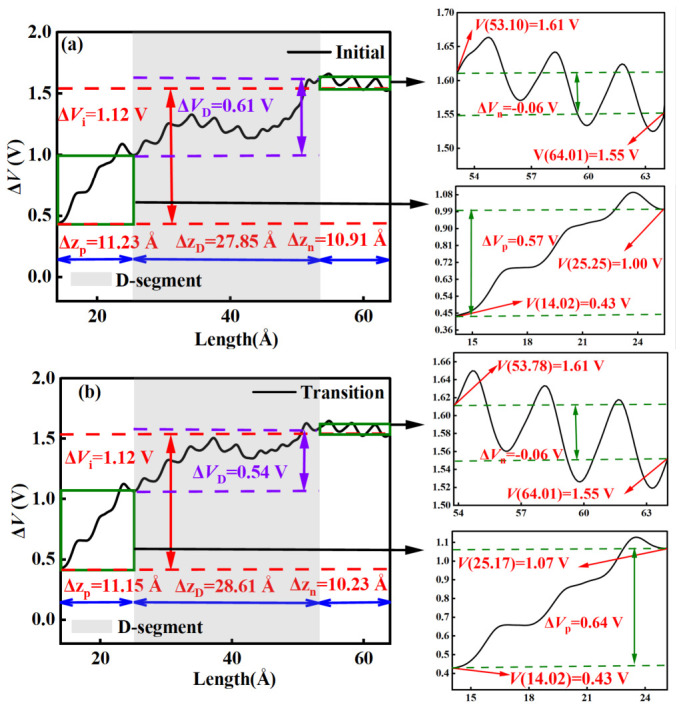

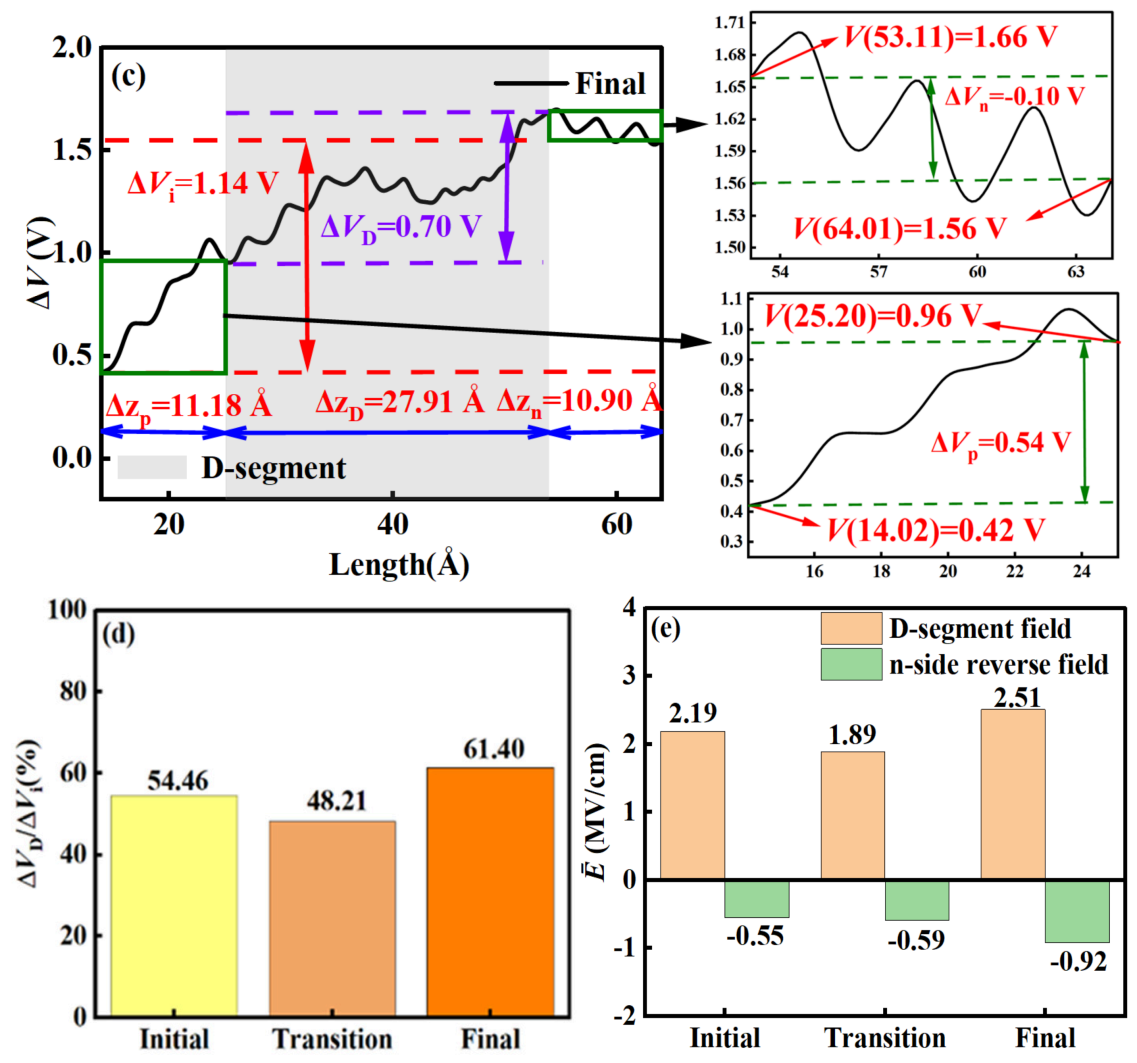
Spatial redistribution of the built-in electrostatic potential in DW-containing devices. Built-in potential profiles for the (**a**) Initial, (**b**) Transition, and (**c**) Final states are shown along the transport direction. The channel is divided into the p-side segment, the DW-containing D segment, and the n-side segment. (**d**) Fraction of the total potential drop carried by the D segment, Δ*V*_D_/Δ*V*_i_. (**e**) Average driving field in the D segment and reverse field in the n-side segment.

After photoexcitation, carrier separation is governed by the spatial distribution of the built-in potential (Δ*V*) within the intrinsic region. As illustrated in Figure 11, the channel is divided into three segments along the transport direction: the p-side segment near the p electrode (Δ*z*_p_), the DW-containing segment (Δ*z*_D_), and the n-side segment near the n electrode (Δ*z*_n_). The average electric field in each segment is given by *Ē* = Δ*V*/Δ*z*.

The total potential drop across the channel remains nearly identical for all configurations (Initial: 1.12 V, Transition: 1.12 V, Final: 1.14 V), with differences below 2%. This confirms that the overall electrostatics is dominated by doping-induced screening. Because the total potential drop is similar for all DW-containing devices, the key difference lies not in the magnitude of the overall built-in field but in how the potential drop is spatially redistributed among the p-side, DW-containing, and n-side segments. In other words, the spatial distribution of this potential drop becomes strongly configuration-dependent once a DW is introduced.

For the Initial state (Figure 11a), the DW-containing segment carries a potential drop of Δ*V*_D_ = 0.61 V (54.46% of the total), corresponding to an average field of *Ē*_D_ = 2.19 MV·cm^−1^. The n-side segment exhibits a weak reverse field of *Ē*_n_ = −0.55 MV·cm^−1^. In the Transition state (Figure 11b), a large fraction of the potential drop shifts to the p-side segment (Δ*V*_p_ = 0.64 V, 57.14% of the total), reducing the effective field in the DW region to *Ē*_D_ = 1.89 MV·cm^−1^. For the Final state (Figure 11c), the DW segment carries the largest potential drop (Δ*V*_D_ = 0.70 V, 61.40%), resulting in the strongest driving field (*Ē*_D_ = 2.51 MV·cm^−1^). However, this is accompanied by a significantly stronger reverse field in the n-side segment (*Ē*_n_ = −0.92 MV·cm^−1^).

A comparison of the three configurations reveals two key trends (Figure 11d,e). First, the driving field in the DW region follows the order Final > Initial > Transition. Second, the reverse field in the n-side segment is substantially larger in the Final state than in the other two configurations. This strong reverse field partially offsets the benefit of the enhanced driving field by pulling carriers back toward the channel.

Consequently, the Initial state achieves the most favorable balance between an efficient driving field for carrier separation and minimal reverse-field suppression, leading to the highest carrier-separation efficiency among the three configurations.

(iii) Carrier-extraction efficiency: quantum connectivity of the conduction band

The analysis in Section 3.2.1 demonstrated that the spatial connectivity of conduction-band states plays a decisive role in carrier extraction. In DW-containing devices, however, the situation becomes more complex. A DW, as a local structural discontinuity, partitions the channel into segments with opposite polarization and distinct local band structures. In addition, the DW itself may introduce localized bound states. As a result, photogenerated carriers must traverse multiple segments sequentially, and the overall extraction efficiency is governed by the weakest link in this series transport pathway.

To avoid relying only on visual inspection of the LDOS maps, we further quantify the spatial continuity of conduction-band states using an energy-integrated LDOS profile and a bottleneck ratio. Specifically, we define an energy-integrated spatial distribution function, *I*(*z*; E*), by integrating the LDOS over an energy window centered at the dominant target energy E*:

(6) $$I\left(z;E^{*}\right)=\int_{E^{*}-\Delta E}^{E^{*}+\Delta E}LDOS\left(E,z\right)dE\;$$

Here, E* is chosen as the conduction-side DOS peak associated with the dominant optical transition for each configuration (Initial: 1.08 eV; Transition: 1.00 eV; Final: 2.45 eV). The rationale is that carriers contributing most to the photocurrent are excited into conduction-band states near E*, so the spatial continuity of LDOS at this energy directly reflects the efficiency of carrier extraction.

An energy window of 2Δ*E* (0.36 eV) is adopted to capture low-energy states near the conduction-band edge while excluding higher-energy contributions. To isolate intrinsic transport within the channel, several non-intrinsic regions are excluded: (i) the p/n electrode regions, (ii) the electrode–channel interfacial regions (1.5 Å at each boundary), and (iii) the DW cores and their adjacent transition regions (±1.5 Å), which contain localized states that do not contribute to long-range transport. We verified that varying the exclusion width (0–3 Å) does not affect the ranking or the order-of-magnitude differences among the configurations.

**Figure 12:**
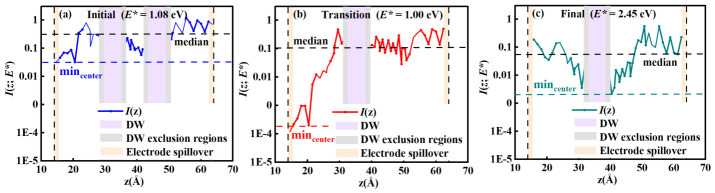
Spatial distribution of the energy-integrated local density of states, *I*(*z*; E*), along the transport direction for the three domain-wall configurations at their respective conduction-band minima: (**a**) Initial state (E* = 1.08 eV), (**b**) Transition state (E* = 1.00 eV), and (**c**) Final state (E* = 2.45 eV). E* corresponds to the conduction-band minimum peak in the DOS of each state (see Figure 10). Line plots represent *I*(*z*; E*)after excluding electrode penetration and domain-wall-localized states. Shading conventions: purple for domain-wall regions, gray for excluded domain-wall regions including ±1.5 Å transition zones at both ends, and orange for electrode wavefunction penetration regions (1.5 Å at the ends of the intrinsic region).

After these exclusions, the effective analysis regions differ among the three configurations due to the number of DWs present. The Initial state contains two DWs and retains ~59.8% of the channel as a clean transport region, whereas the Transition and Final states contain one DW and retain ~76.8% and ~77.2%, respectively. Figure 12 presents the spatial distribution of *I*(*z*; E*) on a logarithmic scale for all configurations.

The Initial state exhibits remarkable spatial uniformity at E* = 1.08 eV (Figure 12a). The variation in *I*(*z*; E*) remains within approximately one order of magnitude across the channel, corresponding to a bottleneck ratio of *C* ≈ 9.60. Such weak fluctuations are insufficient to form an effective scattering barrier. Consequently, electronic wave functions remain spatially coherent, enabling carriers to propagate smoothly from the absorption region to the electrode with minimal loss.

In contrast, the Transition state (E* = 1.00 eV) exhibits a pronounced transport bottleneck. In the region *z* ≈ 15.5–31.0 Å, *I*(*z*; E*) drops to ~10^−4^ (min_center_ = 2.02 × 10^−4^), more than two orders of magnitude lower than in the Initial state. This indicates a near absence of accessible conduction-band states, effectively suppressing quantum transport. Although *I*(*z*; E*) recovers in the downstream region, carriers must traverse this low-density region, resulting in exponentially suppressed transmission and severely limited extraction efficiency.

The Final state (E* = 2.45 eV) shows intermediate connectivity. Its minimum value (min_center_ = 2.22 × 10^−3^) is higher than that of the Transition state but remains lower than that of the Initial state. Moreover, its higher evaluation energy implies that carriers must be excited to higher energies to access conduction-band states with sufficient spatial continuity. This explains the observed shift in the photocurrent peak toward higher photon energy (4.39 eV).

To quantify spatial continuity, we define the bottleneck ratio *C* = median_full_/min_center_, which measures the contrast between typical and minimum state densities. Larger *C* values indicate more severe transport bottlenecks. The three configurations yield *C* ≈ 9.6 (Initial), 508 (Transition), and 28.2 (Final), consistent with the trends observed in the *I*(*z*; E*) profiles.

These results demonstrate that conduction-band connectivity is the decisive factor governing carrier extraction. The Initial state maintains near-continuous electronic pathways, whereas the Transition state introduces a pronounced transport barrier, and the Final state exhibits intermediate behavior.

As summarized in Table 1, the Initial state achieves the highest photocurrent not because it has the strongest individual separation field, but because it provides the best overall balance among transition-relevant DOS, carrier-separation field distribution, and conduction-channel connectivity.

(iv) Integrated discussion: joint action of multiple factors

Taking these three factors together (Table 1), the superior photoelectric performance of the Initial state does not originate from an absolute advantage in any single factor. For example, the Final state exhibits the strongest driving field in the D segment. Instead, the key advantage of the Initial state lies in its best overall balance among the three factors. It possesses the most favorable DOS pairing on both sides of the dominant transition, concentrates a sufficiently large built-in potential drop in the D segment while maintaining the weakest reverse field in the n-side segment, and preserves the highest effective spatial continuity of conduction-band states along the transport direction under the fixed device orientation (*C* ≈ 9.6). This connectivity advantage arises from the combined effect of polarization-dependent configuration changes and the associated local structural reconstruction. As a result, the Initial state is most favorable for maintaining electronic-state coupling along the transport direction, thereby supporting the highest peak photocurrent (10.91 μA·mm^−2^).

The Transition state shows the opposite behavior, with all three factors acting as bottlenecks. The density of final conduction-band states is less than one-third of that in the Initial state, which severely restricts the available transition channels. In addition, the potential drop is shifted toward the p-side segment, meaning that the driving force for carrier separation is consumed outside the core photoactive region. Most importantly, *I*(*z*; E*) in the channel region extending from the p-side segment to the left side of the DW decreases to the 10^−4^ level (*C* ≈ 508), indicating extremely poor effective conduction-band connectivity along the transport direction. In other words, under the present crystal orientation and device geometry, the local atomic arrangement of this transition configuration, together with its polarization-coupled response, fails to maintain a continuous electronic propagation pathway. Carrier transmission is therefore strongly suppressed in this region, and the combined limitations of all three factors result in the weakest photocurrent (5.92 μA·mm^−2^).

The Final state is characterized by a strong driving field accompanied by a strong reverse field. Although the driving field in the D segment is the largest among the three configurations, the reverse field in the n-side segment is about 55.93% larger than that in the Initial state. Consequently, electrons that have already been separated experience a stronger pullback before reaching the n electrode, partially offsetting the benefit of the stronger separation field. In terms of carrier extraction, the effective conduction-band connectivity of the Final state (*C* ≈ 28.2) is better than that of the Transition state but still inferior to that of the Initial state. This indicates that, although the Final state can maintain a certain degree of continuous propagation, the coupling of electronic states along the transport direction remains weaker than in the Initial state. Moreover, its dominant transition target lies in a higher-energy region, and its optical absorption remains at an intermediate level. Together, these factors place the Final state’s peak photocurrent in second position (8.39 μA·mm^−2^). Finally, it must be noted that the present NEGF calculations evaluate the ballistic transport limit at 0 K. In practical devices operating at finite temperatures, thermal effects will influence both domain wall stability and optoelectronic transport. Thermodynamically, the switching activation barrier of 0.37 eV is physically sufficient to guarantee that the static DW configurations persist in the channel at room temperature. However, finite temperatures inevitably introduce electron–phonon scattering, which disrupts wave-function coherence and reduces the carrier mean free path. Consequently, while the configuration-selective enhancement mechanisms—driven by built-in potential redistribution and density of states connectivity—are expected to hold qualitatively, the absolute photocurrent densities in room-temperature devices will be lower than the theoretical ballistic limits predicted in this study.

## 4. Conclusions

As device dimensions continue to shrink, the fraction of the active channel occupied by domain walls becomes increasingly significant. We emphasize that the present calculations are performed for a 50 Å intrinsic channel, in which the domain-wall region occupies a non-negligible fraction of the active device. Under this short-channel condition, certain thermodynamically stable domain-wall configurations can evolve from passive scattering centers into functional interfaces that actively enhance photocurrent. For longer channel lengths, however, at fixed domain-wall number and fixed local domain-wall width, the fractional contribution of the DW-modulated region is expected to decrease. In this limit, the DW-induced redistribution of the built-in potential and the associated modulation of LDOS connectivity should evolve from a channel-wide effect into a more localized perturbation. Accordingly, the enhancement is expected to persist qualitatively as a local configuration-dependent effect, while its quantitative impact on the total photocurrent should decrease with increasing channel length, causing the device response to move toward that of the corresponding single-domain reference. Establishing the exact scaling law with channel length is beyond the scope of the present work. Importantly, the enhancement remains strongly configuration-dependent, providing physically grounded criteria for domain-wall engineering in short-channel low-dimensional optoelectronic devices.

Using first-principles quantum-transport calculations on monolayer ferroelectric ZB′-In_2_Se_3_ p–i–n junctions, we benchmarked the two single-domain configurations (ABBCA and ABCCA). We attributed the difference in their photocurrents to the combined effects of density of states and conduction-band connectivity. We then introduced two thermodynamically stable DW configurations along the polarization-reversal pathway (the Initial and Final states) into the device channel, using the saddle-point configuration (the Transition state) as a reference. Contrary to the conventional expectation that DWs act as scattering centers that degrade device performance, both stable DW configurations substantially enhance the photocurrent. The Initial state reaches 10.91 μA·mm^−2^, approximately 1.80 times that of the single-domain device, while the Final state reaches 8.39 μA·mm^−2^, corresponding to an enhancement of ~37%. In contrast, the Transition state (5.92 μA·mm^−2^) remains comparable to the single-domain benchmark and shows no appreciable enhancement, demonstrating clear configuration selectivity.

Examining the configuration-dependent trends in optical absorption, carrier separation, and carrier extraction (which are coupled in the self-consistent NEGF framework) provides a qualitative explanation for the performance differences. The three-factor analysis is a post hoc qualitative rationalization of self-consistent NEGF quantities, not a unique causal decomposition of independent physical effects. It serves to illustrate how different regions of the device contribute to the final photocurrent, but the reported photocurrent values are obtained from full NEGF without any decoupling. The superiority of the Initial state arises from the most favorable overall balance among these three factors: it provides the most favorable pairing of valence-band and conduction-band DOS for the dominant optical transition, a sufficiently large potential drop in the D segment together with the weakest reverse field in the n-side segment, and spatially continuous conduction-band states across the channel. In the Transition state, all three factors become limiting: the density of final conduction-side states is insufficient, the separation-driving field is shifted toward the p-side segment, and the conduction-band connectivity is severely disrupted. The Final state exhibits a strong driving field but also a strong reverse field, which partially offsets the gain in carrier separation. This three-factor balance offers experimentally testable guidance: in the present 50 Å short-channel model, thermodynamically stable DW configurations (e.g., the Initial state) are expected to yield photocurrents up to 1.80 times that of the corresponding single-domain reference, providing a clear target for domain-wall engineering in short-channel 2D ferroelectric optoelectronics.

## Figures and Tables

**Table 1:** Comparison of the three governing factors for the three DW configurations.

Factor	Key Descriptor	Initial	Transition	Final
Absorption	Transition-relevant DOS	Pronounced peak at E* ≈ 1.08 eV	Weak peak at E* ≈ 1.00 eV	High-energy dominant transition at E* ≈ 2.45 eV
Separation (driving field)	*E*_D_ in the D segment	2.19 MV·cm^−1^	1.89 MV·cm^−1^	2.51 MV·cm^−1^
Separation (reverse field)	Reverse field in n region	−0.55 MV·cm^−1^	−0.59 MV·cm^−1^	−0.92 MV·cm^−1^
Extraction (effective connectivity)	Bottleneck ratio *C*	9.6 (best)	508 (worst)	28.2 (intermediate)
Photocurrent	*J*_ph,y_ (μA·mm^−2^)	10.91	5.92	8.39

## Data Availability

The data presented in this study are available on request from the corresponding author.
